# The Ellis County Medical Society

**Published:** 1885-07

**Authors:** 


### Society Notes.

The Ellis County Medical Society has a membership of thirty-five and "it is nothing uncommon to find them all present during the sessions of the society," says the accomplished and progressive Secretary Dr. R. B. White. They meet at Waxahachie monthly, and every physician in that city is a member. Here is an example worthy of imitation. It is one of the results of journalistic influence.

